# Validity of an inertial sensor-based system for the assessment of spatio-temporal parameters in people with multiple sclerosis

**DOI:** 10.3389/fneur.2023.1164001

**Published:** 2023-04-20

**Authors:** Annalena Zahn, Veronika Koch, Lucas Schreff, Patrick Oschmann, Jürgen Winkler, Heiko Gaßner, Roy Müller

**Affiliations:** ^1^Department of Neurology, Klinikum Bayreuth GmbH, Bayreuth, Germany; ^2^Department of Molecular Neurology, University Hospital Erlangen, Friedrich-Alexander-University Erlangen, Erlangen, Germany; ^3^Fraunhofer Institute for Integrated Circuits (IIS), Digital Health Systems, Erlangen, Germany; ^4^Bayreuth Center of Sport Science, University of Bayreuth, Bayreuth, Germany

**Keywords:** multiple sclerosis (MS), wearable sensors, gait-parameter, walking human, three-dimensional gait analysis, different walking speeds

## Abstract

**Background:**

Gait variability in people with multiple sclerosis (PwMS) reflects disease progression or may be used to evaluate treatment response. To date, marker-based camera systems are considered as gold standard to analyze gait impairment in PwMS. These systems might provide reliable data but are limited to a restricted laboratory setting and require knowledge, time, and cost to correctly interpret gait parameters. Inertial mobile sensors might be a user-friendly, environment- and examiner-independent alternative. The purpose of this study was to evaluate the validity of an inertial sensor-based gait analysis system in PwMS compared to a marker-based camera system.

**Methods:**

A sample *N* = 39 PwMS and *N* = 19 healthy participants were requested to repeatedly walk a defined distance at three different self-selected walking speeds (normal, fast, slow). To measure spatio-temporal gait parameters (i.e., walking speed, stride time, stride length, the duration of the stance and swing phase as well as max toe clearance), an inertial sensor system as well as a marker-based camera system were used simultaneously.

**Results:**

All gait parameters highly correlated between both systems (*r* > 0.84) with low errors. No bias was detected for stride time. Stance time was marginally overestimated (bias = −0.02 ± 0.03 s) and gait speed (bias = 0.03 ± 0.05 m/s), swing time (bias = 0.02 ± 0.02 s), stride length (0.04 ± 0.06 m), and max toe clearance (bias = 1.88 ± 2.35 cm) were slightly underestimated by the inertial sensors.

**Discussion:**

The inertial sensor-based system captured appropriately all examined gait parameters in comparison to a gold standard marker-based camera system. Stride time presented an excellent agreement. Furthermore, stride length and velocity presented also low errors. Whereas for stance and swing time, marginally worse results were observed.

## 1. Introduction

Multiple sclerosis (MS) is the most frequent progressive neurological disease of the central nervous system affecting young adults (1). It is caused by an inflammatory process in the myelin sheaths of the brain and spinal cord, which leads to demyelinating of the nerval axons and impairment of the nerve cell function (2). This results in various symptoms including impaired walking leading to a negative impact on the patient's quality of life (3).

Even in early stages without clinical signs of walking disability, gait and balance impairments were observed (4–9). Previous findings in motor impaired people with MS (PwMS) were reduced stride length and gait speed as well as an elevated stride time and stance time (4–7, 9, 10). Those gait parameters correlated with the disease-associated fatigue and may be used to define the outcome of pharmaceutical trials (6, 7, 9, 11). Furthermore, gait parameters can be associated with a patients' fall risk. Scholz et al. summarized that PwMS who fell more frequently had lower walking speed, shorter stride length and worse balance (12).

At present, the gold standard to capture gait parameters of patients with neurological disorders are optical marker-based camera systems (4, 13). They are known to mirror the human gait very precisely, but require a lot of time, cost, and knowledge for performing and interpreting a study. Furthermore, the gait analysis is limited to specialized centers, fixed appointments and could not be performed in the patients' domestic environment and at flexible times. Inertial mobile sensors have been proven to be a valid alternative and could even replace laboratory gait analysis systems in terms of portability and offering continuous records without constraining topical and temporal limitations (14, 15). Among others, Moufawad el Achkar et al. (16) presented that foot-worn inertial sensors are valid to monitor the activity and gait of older people for an extended time span and in non-clinical settings.

Mobile inertial sensors have already been used to examine gait in different neurological diseases. A systematic review has proven the clinical validity of inertial sensor systems for neurological disorders (17). Sensor-based gait analysis was for example used to objectively measure gait disturbances in Parkinson's disease and atypical parkinsonian disorders (18, 19) and to quantify disease severity and impairment in Huntingtons's disease (20, 21). Furthermore, the findings of gait variability in PwMS, which were at first reported in laboratory-based gait analysis (4, 13), were also replicated in more recent studies with mobile inertial sensors. They were reported to be valid and reliable sources to assess gait abnormalities even in milder stages of MS (5, 6). Moreover, Angelini et al. (22) confirmed the reliability of inertial sensors in gait analysis by recording gait in PwMS in two different hospitals and under different gait testing conditions. Additionally, Vivienne-Jumeau et al. (23) showed in a systematic review, that inertial sensors provide reliable data for estimation of disease severity in PwMS by comparing inertial sensors to disease severity scales. In our study, we aim to complement those findings by contributing to the hypothesis, that those inertial sensors are technically robust and precise in PwMS by comparing sensor-derived outcomes to those of a camera-based system. Kluge et al. already compared those inertial foot-worn sensors to a reference camera-based motion capture system for healthy participants and patients with Parkinson's disease and showed a valid agreement between both systems for all gait parameters (24, 25). Other researchers likewise investigated the accuracy of inertial sensors by comparing them to a camera-based system. They were proven to be a valid tool to detect walking cadence and postural responses in PwMS (26, 27). Nonetheless, current study situations miss this comparison of spatio-temporal gait parameters derived from foot-worn inertial sensor systems and camera-based systems in PwMS.

In this study, we further elaborate the validity of inertial sensors in PwMS and healthy participants. To this end, clinically interpretable spatio-temporal gait parameters were collected using inertial mobile sensors and a state-of-the-art marker-based camera system. We hypothesized that the gathered data between both systems have no significant difference hence showing that mobile sensor systems precisely analyze gait parameters of PwMS.

## 2. Materials and methods

### 2.1. Participants

Thirty-nine PwMS and 19 healthy age- and gender-matched participants were recruited in the Department of Neurology of the Klinikum Bayreuth GmbH, Germany. Table 1 presents the demographic and clinical characteristics of the study participants. PwMS implemented the gait analysis in connection to their routine care or during their inpatient stay. Inclusion criteria consisted of a verified MS diagnosis (28), an age between 18 and 65 years and the ability to walk without a walking aid for at least 10 m. Healthy participants declared to have no conditions that might influence gait such as neurological or orthopedic disorders. All participants provided written informed consent prior to the first study visit. The study was approved by the ethical committee of the University Hospital Erlangen (166_18 B) and was in accordance with the Declaration of Helsinki.

**Table 1:** Demographical and clinical characteristics of the study participants.

	**Healthy participants**	**PwMS**	***p*-value**
Participants number	19	39	
Female sex	12	29	0.666
Age (years)	43.4 ± 14.9	43.7 ± 11.5	0.916
Height (cm)	171.8 ± 8.3	170.5 ± 7.2	0.549
Weight (kg)	72.1 ± 16.4	77.5 ± 20.6	0.313
EDSS	NA	2.8 ± 1.3	
**Type of MS**			
Relapsing-remitting	NA	29	
Secondary progressive	NA	10	

Values of age, height, weight and EDSS are expressed as mean ± SD.

MS, multiple sclerosis; EDSS, expanded disability status scale.

### 2.2. Measurements

Assessments were conducted in a laboratory setting in the Klinikum Bayreuth GmbH, Department of Neurology Bayreuth, Germany. The participants were required to walk a straight and even-floored distance of 10 m six times at three different self-selected walking speeds (fixed order: normal, fast, slow) to cover a wide range of various walking speeds. In our experimental design we adjusted the methods of Kluge et al. (24), who validated a similar sensor-based system in patients with Parkinson's disease.

**Figure 1:**
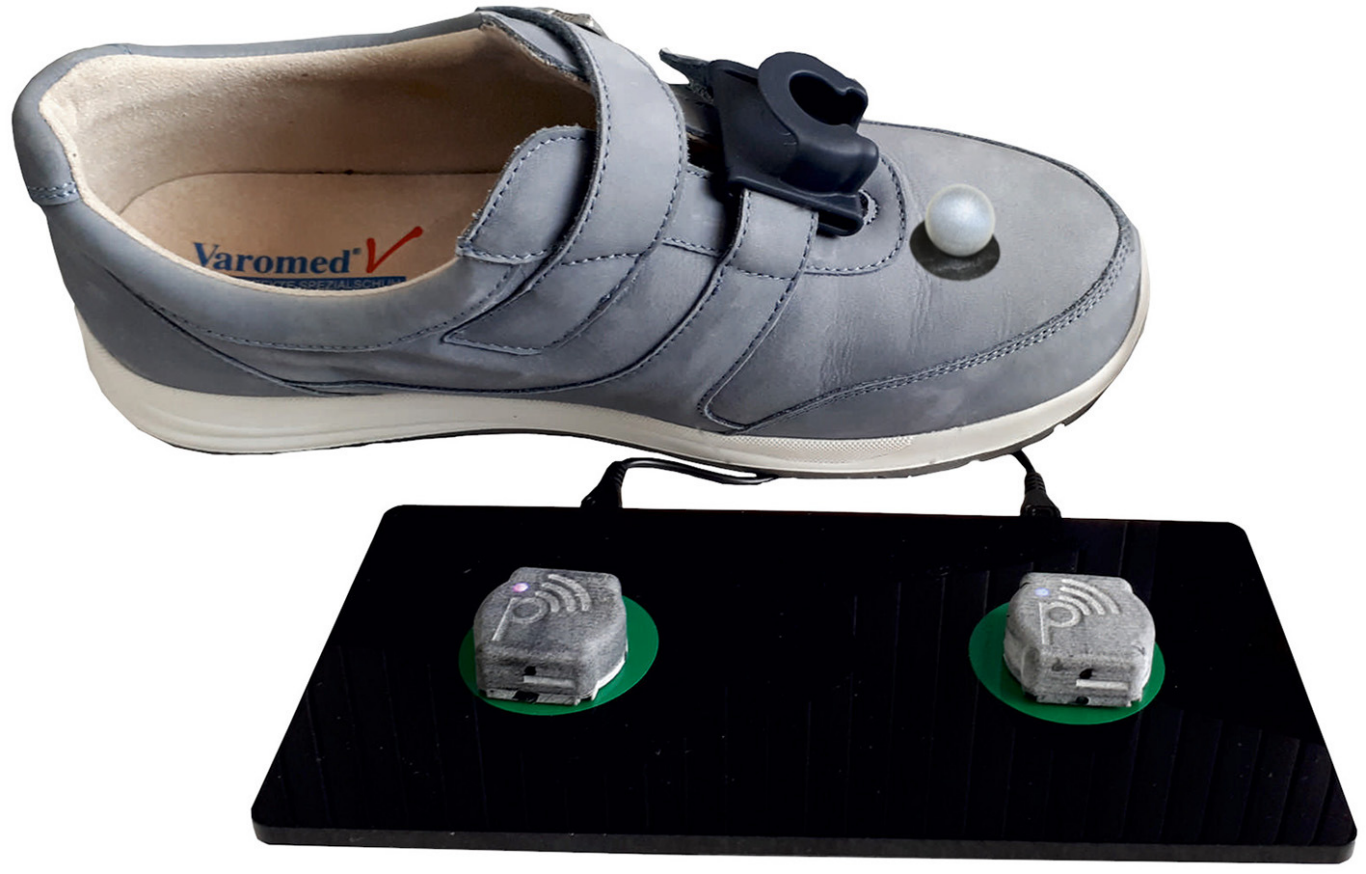
The inertial sensors were attached to the instep and the reflective markers were placed on the head of the second metatarsal bone on both right and left shoe.

To measure spatio-temporal gait parameters (i.e., stride time, stride length, walking speed the duration of the stance and swing phase as well as max toe clearance), an inertial sensor system (Mobile GaitLab, PHCT GmbH, Erlangen, Germany) as well as a marker-based camera system (VICON, Oxford, UK) were used simultaneously. The inertial sensor system consists of two inertial sensors, secured to the instep of each shoe of both participants' feet (Figure 1). Each inertial measurement unit consists of a 3D-accelerometer (range ±16 g) measuring linear acceleration as well as a 3D-gyroscope (range ± 2,000 deg/s) measuring angular velocity (rate of change of angle). The sampling rate was set to 102.4 Hz and the raw data was streamed wirelessly via Bluetooth^®^ to an android tablet, transferred and stored on a computer for further analysis. Additionally, reflective markers (16 mm) were placed on the head of the second metatarsal bone on both right and left foot (Figure 1). Both, the camera system, and the inertial mobile sensor system were synchronized manually by a quick stamp on the ground similar to Uno et al. (29) who used a vertical jump at the beginning of each trial. The measurements, including the placement of the markers and inertial sensors, have been performed by the same researchers, ensuring conformity.

### 2.3. Data processing

To assure that the subjects walked at a steady speed and to exclude turning, initiation or stopping strides, solely one left and one right stride in the middle of the walkway over the force plates were used. All spatio-temporal gait parameters were calculated twice, once via the Software of the 3D camera system (Vicon Nexus, Vicon ProCalc) and once via validated algorithms for the mobile sensor system (30, 31).

### 2.4. Statistical analysis

Statistical analyses were performed using SPSS 20 (Chicago, IL, USA). Participant characteristics were compared using Pearson's Chi-square for gender and independent *t*-Tests for age, height, and weight. Of the healthy participants, 688 strides were used for the analysis, while the remaining 1,324 strides originated from PwMS. We assessed concurrent validity of all spatio-temporal gait parameters by calculating Pearson's correlation, bias (mean difference), absolute error and the relative absolute error as agreement measures between both the camera and the sensor system. We used all single strides (*n* = 2,012) from all speeds (normal, fast, slow) in the validity analysis to cover a large range of gait parameters. Furthermore, Bland-Altman plots visualize the mean of the difference (bias) as well as the 95% confidence interval of the bias between the two systems (32). Validity between both systems was also assessed for healthy participants and PwMS to evaluate whether mildly affected gait would affect the system's accuracy. Differences were assessed by a one-way ANCOVA with group (healthy participants, PwMS) as factor and speed (normal, slow, fast) as covariate. An alpha level of 0.05 was used for all statistical tests.

## 3. Results

**Figure 2:**
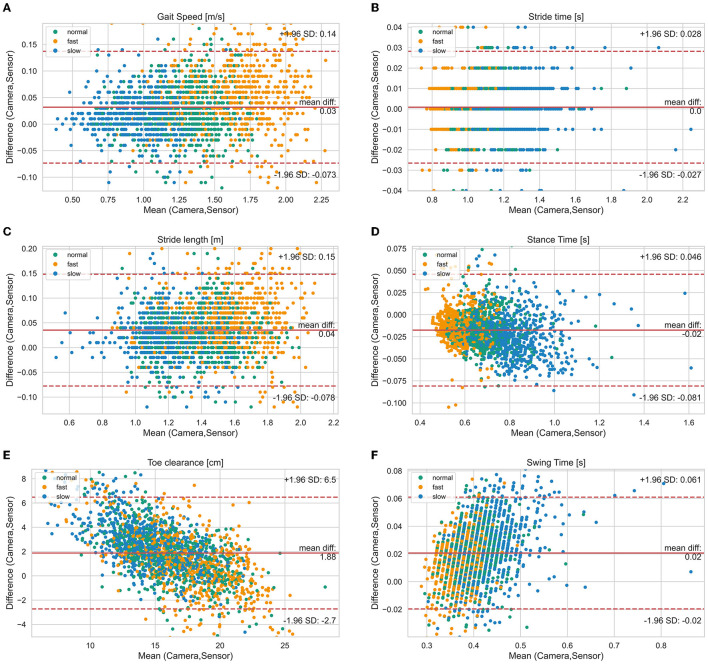
Bland-Altman diagrams of gait parameters [**(A)** gait speed, **(B)** stride time, **(C)** stride length, **(D)** stance time, **(E)** toe clearance, **(F)** swing time] show the difference vs. the mean of both systems for all single strides. The solid line indicates the bias and the dashed lines the limits of agreement (95% confidence interval of the bias). Highlighted by colors are the three different test speeds (normal, slow, fast).

Mean ± SD values of all measured spatio-temporal gait parameters together with agreement measures are given in Table 2. High correlations (*r* > 0.84) with low errors were observed for all gait parameters (Table 2). While no bias was observed for stride time, stance time was slightly overestimated and swing time underestimated, respectively, by about 0.02 s (Figure 2). The stride length was underestimated by 0.04 m, the gait speed was underestimated by 0.03 m/s and max toe clearance was underestimated by 1.88 cm. The absolute relative error of the sensor-based gait parameters was below 14.1% for all gait parameters. The results of the parameters involving temporal information showed an error between 0.9% (stride time) and 4.9% (swing time). The results of the parameters involving spatial information showed an error between 3.5% (stride length) and 14.1% (max toe clearance). Table 3 differentiates the results of the spatio-temporal gait parameters between healthy participants and PwMS.

**Table 2:** Overview of spatio-temporal gait parameters for 38 PwMS and 19 healthy participants (*n* = 2012 strides).

	**Camera**	**Sensor**	***r***	**Bias**	**Abs. error**	**Error (%)**
Gait speed (m/s)	1.33 ± 0.37	1.30 ± 0.36	0.99	0.03 ± 0.05	0.05 ± 0.04	3.8
Stride length (m)	1.41 ± 0.26	1.38 ± 0.25	0.98	0.04 ± 0.06	0.05 ± 0.05	3.5
Stride time (s)	1.10 ± 0.18	1.10 ± 0.18	1.00	0.00 ± 0.02	0.01 ± 0.01	0.9
Stance time (s)	0.69 ± 0.13	0.70 ± 0.14	0.97	−0.02 ± 0.03	0.02 ± 0.03	2.9
Swing time (s)	0.41 ± 0.06	0.39 ± 0.05	0.94	0.02 ± 0.02	0.02 ± 0.02	4.9
Toe clearance (cm)	17.0 ± 2.3	15.1 ± 4.1	0.84	1.88 ± 2.35	2.39 ± 1.82	14.1

Shown are the mean parameters ± SD, Pearson correlation coefficient r, bias ± SD, absolute error ± SD and the relative absolute error.

**Table 3:** Overview of spatio-temporal gait parameters for 38 PwMS and 19 healthy participants (*n* = 2012 strides).

	**Camera**	**Sensor**	***r***	**Bias**	**Abs. error**	**Rel. error**
**Gait speed (m/s]**						
PwMS	1.26 ± 0.34	1.23 ± 0.33	0.99	0.03 ± 0.05	0.05 ± 0.04	4.0
Healthy	1.45 ± 0.38	1.42 ± 0.36	0.99	0.04 ± 0.06	0.05 ± 0.05	3.4
**Stride length (m]**						
PwMS	1.35 ± 0.25	1.32 ± 0.24	0.97	0.03 ± 0.06	0.05 ± 0.05	3.7
Healthy	1.53 ± 0.24	1.49 ± 0.22	0.98	0.04 ± 0.05	0.05 ± 0.04	3.3
**Stride time (s]**						
PwMS	1.10 ± 0.18	1.10 ± 0.18	1	0.00 ± 0.02	0.01 ± 0.01	0.9
Healthy	1.09 ± 0.17	1.09 ± 0.17	1	0.00 ± 0.01	0.01 ± 0.01	0.9
**Stance time (s]**						
PwMS	0.69 ± 0.14	0.71 ± 0.14	0.96	−0.02 ± 0.04	0.03 ± 0.03	4.3
Healthy	0.67 ± 0.13	0.69 ± 0.13	0.99	−0.02 ± 0.02	0.02 ± 0.01	3.0
**Swing time (s]**						
PwMS	0.41 ± 0.06	0.39 ± 0.04	0.96	0.02 ± 0.02	0.02 ± 0.02	4.9
Healthy	0.42 ± 0.05	0.40 ± 0.05	0.95	0.02 ± 0.02	0.03 ± 0.02	7.1
**Toe clearance (cm]**						
PwMS	16.6 ± 2.6	14.4 ± 4.0	0.81	2.21 ± 2.44	2.62 ± 2.00	15.8
Healthy	17.7 ± 2.8	16.5 ± 3.9	0.88	1.23 ± 2.02	1.96 ± 1.33	11.1

Presented are the mean parameters ± SD, Pearson correlation coefficient r, bias ± SD, absolute error ± SD and the relative absolute error.

**Figure 3:**
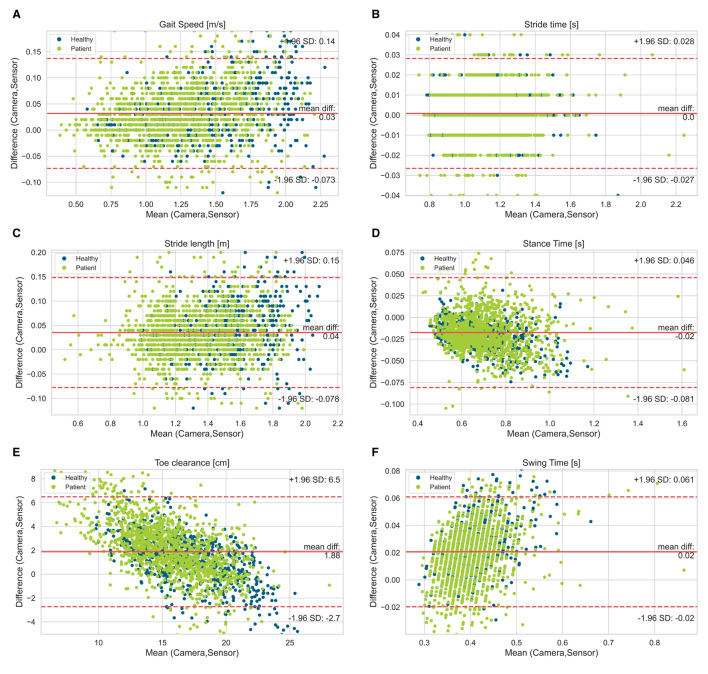
Bland-Altman diagrams of gait parameters [**(A)** gait speed, **(B)** stride time, **(C)** stride length, **(D)** stance time, **(E)** toe clearance, **(F)** swing time] show the difference vs. the mean of both systems for all single strides. The solid line indicates the bias and the dashed lines the limits of agreement (95% confidence interval of the bias). Highlighted by colors are the healthy participants and patient group (PwMS).

Figure 2 visualizes the agreement between both systems regarding different walking speed conditions and Figure 3 visualizes the agreement between both systems regarding both healthy participants and PwMS. The bias of gait speed [*F*_(1, 1981)_ = 12.58, *p* = 0.000], stance time [*F*_(1, 1997)_ = 17.91, *p* = 0.000), swing time [*F*_(1, 1998)_ = 31.38, *p* = 0.000] and max toe clearance [*F*_(1, 1999)_ = 89.14, *p* = 0.000] differ between healthy participants and PwMS. However, these differences were also significantly affected by the covariate speed. The bias of stride time [*F*_(1, 1982)_ = 2.35, *p* = 0.126] and stride length [*F*_(1, 1998)_ = 2.87, *p* = 0.091] did not differ between healthy participants and PwMS.

## 4. Discussion

According to this study, a mobile sensor system provides valid information about spatio-temporal gait parameters of healthy participants and PwMS. The gait parameters of the sensor system have good conformity with the gait parameters of the camera system. The collected spatio-temporal gait parameters were also comparable to the established results in former studies concerning healthy adults and PwMS (5–7, 9, 13), indicating that their gait pattern was measured correctly.

Stride time presented an excellent agreement. Furthermore, stride length and velocity presented also low errors. Whereas for stance time and swing time marginally worse results were observed. On average, the swing time was underestimated, and the stance time was overestimated by the inertial sensor systems resulting in a percentage longer stance phase measured in the gait cycle. This might be caused by the different measurements of the heel strike and toe-off events, which separate the stance phase from the swing phase. The camera system used to define the participants' heel strike by the pressure the touchdown put on a sensitive force plate, which detected the heel strike already at a force threshold of 10 N. Whereas the inertial mobile sensors detect the end of the swing phase through the change from dorsal extension to plantar flexion of the foot resulting in a longer duration of the swing time and consecutive in a shorter stance time. These findings correspond also approximately with the comparison of a camera system and those inertial sensors measuring patients with Parkinson's disease (24).

The worst but nevertheless good agreement between both systems showed the max toe clearance with a Pearson correlation of 0.81 in PwMS and 0.88 in healthy participants. This might be based on the fact, that the max toe clearance was defined differently. While the camera system calculated the distance between the maximal and minimal height of the toe marker position, the mobile sensor system measured the height above the ground and used the shoe size (defined shoe length for each size) to calculate the max toe clearance. Furthermore, the inertial sensor was placed distal (depending on the type of the individual shoe) of the reflective marker (Figure 1); hence there might be a difference in the absolute distance because of the range of the extension and flexion in the ankle joint.

The bias of the gait speed and stride time deviated between the healthy participants and the PwMS by having slightly lower bias for the participants affected by MS. This could be an effect of the result that gait speed and stride time were significantly affected by the covariate speed, which is also shown in the Bland-Altman plots. Consequently, bias measured in a fast walk was higher than the bias detected in a slow walk. On average, PwMS walk slower than the healthy participants, which is also shown in various former studies. This leads to the suggestion that the inertial sensors detect the gait speed and stride length more exact in slow walkers and therefore in PwMS (4–7, 9, 13).

### 4.1 Limitations of the study

Some limitations of the present study require consideration. First, in our experimental design, we synchronized both the sensor and the camera system manually through a quick stamp at the beginning of every walking course. This can be depicted with the analyzing software of the camera system as well as in the visualized gait waveforms recorded by the inertial sensors. We needed to find the concerned steps manually. An automatic synchronization for better comparison of the steps might be beneficial. Second, we compared selective spatio-temporal gait parameters and did not include gait kinetics and kinematics in this study. Third, our participants were not equally distributed in both genders, but were acquired approximately comparable to the distribution of PwMS in the population with 74% female participants with MS (33). Furthermore, gait was acquired in laboratory setting allowing the acquisition of comparable, reliable, and supervised gait parameters. But the gait shown in a laboratory setting might differ from gait in daily life, especially because of the limited evaluated distances and the visually targeted force plates. Moreover, only straight walking was measured and no turnings acceleration and deceleration or walking on uneven ground were detected. In future studies it should be considered to test the validity of inertial mobile sensors in real-world environments.

## 5. Conclusion

In summary, this study showed that inertial sensor-based systems are a insightful option to analyze spatio-temporal gait-parameters of PwMS and healthy participants. This study contributes to the qualification of inertial sensors to be used in clinical studies e.g., pharmaceutical trials and to define disease severity in MS in medical practice since they represent an user-friendly alternative to quickly collect a large number of data concerning a patient's gait. Given validation in different environmental settings and longer observation periods, this inertial sensor system might be a valid tool to analyze gait parameters of PwMS in real-life settings to monitor therapy effectiveness or evaluate their risk of falling.

## Data availability statement

The raw data supporting the conclusions of this article will be made available by the authors, without undue reservation.

## Ethics statement

The studies involving human participants were reviewed and approved by Ethical Committee of the University Hospital Erlangen 166_18 B. The patients/participants provided their written informed consent to participate in this study.

## Author contributions

AZ, HG, RM, and LS conceived and designed the experiments. AZ recruited participants and drafted the manuscript. AZ, RM, and LS performed the experiments. AZ and RM analyzed the data. VK contributed to the visualization of the sensor data. All authors critically reviewed the manuscript and approved the final version.
